# Structure of Alloys for (Sm,Zr)(Co,Cu,Fe)z Permanent Magnets: II. Composition, Magnetization Reversal, and Magnetic Hardening of Main Structural Components

**DOI:** 10.3390/ma13235426

**Published:** 2020-11-28

**Authors:** Andrey G. Dormidontov, Natalia B. Kolchugina, Nikolay A. Dormidontov, Yury V. Milov, Alexander S. Andreenko

**Affiliations:** LLC “MEM”, 123458 Moscow, Russia; nkolchugina@imet.ac.ru (N.B.K.); ontip@mail.ru (N.A.D.); Milov.yv@mail.ru (Y.V.M.); asa@phys.msu.ru (A.S.A.)

**Keywords:** Sm–Co permanent magnet alloys, magnetic hardening, high-coercivity structure, composition–structure–properties relation

## Abstract

Experimental series of alloys for (Sm,Zr)(Co,Cu,Fe)_Z_ permanent magnets are presented in the concentration ranges that provide wide variations of (4*f*)/(4*d*)/(3*d*) ratios of comprising elements. Optical metallographic analysis, observation of the surface domain structure upon magnetization reversal (Kerr effect), electron microprobe analysis, and measuring the major hysteresis loops of samples at different stages of heat treatment are used to study processes related to the development of the highly coercive state of these samples. It was found that the volume fractions of two main structural components A and B, which comprise 90% of the total sample volume, rigorously control the coercivity at all stages of thermal aging. At the same time, structural components A and B themselves in samples being in the high-coercivity state differ qualitatively and quantitatively in the chemical composition, domain structure and its development in external magnetic fields and, therefore, are characterized by different morphologies of the phases comprising the structural components. Two stages of phase transformations in the sample structure are observed. During isothermal annealing, the cellular structure develops within the B component, whereas, during stepwise (slow) cooling or quenching from the isothermal aging temperature to 400 °C, a phase structure evolves within both the cell boundaries in B and the structural component A. The degree of completion of the phase transformations within micro- and nano-volumes of the components determines the ultimate hysteretic characteristics of the material.

## 1. Introduction

The record temperature stability of characteristics of (Sm,Zr)(Co,Cu,Fe)z permanent magnets determines their traditional applications in a wide range of products. However, the uncertainty in details of the formation of high-coercivity phase structure of these magnets in the course of their heat treatments continues to draw the attention of researchers [1,2,3,4,5].

The high magnetization and the high internal coercive force in the operation temperature range are two the most important characteristics of permanent magnets of any type [6].

Techniques of the powder metallurgy (refinement of an alloy to form magnetically-uniaxial micron-sized particles, alignment of powder in a uniform magnetic field, mechanical compacting in compression molds, and subsequent thermal consolidation or sintering of blanks), which allow one to obtain the maximally possible crystallographic anisotropy of blanks, traditionally are used for manufacturing (Sm,Zr)(Co,Cu,Fe)z permanent magnets. The obtained anisotropic massive with the easy magnetization axes of all grains (crystallites) oriented along single preset direction allows the magnetization potential to be most completely realized [6].

The second essential parameter of permanent magnets is the coercivity, which, in the case of precipitation hardened (Sm,Zr)(Co,Cu,Fe)z alloys, is reached at the expense of the complex heat treatment that consists in the solid-solution heat treatment, quenching, isothermal and stepped aging (or aging during slow cooling). The periodic phase nanostructure results from the sequential phase transformations, and ensures the coercivity mechanism, namely, the efficient domain-wall pinning at interfaces of the formed phases [6].

Powder alloys prepared by the above way demonstrate almost ideal structure of the material and high hysteretic performances.

The homogenization of the (Sm,Zr)(Co,Cu,Fe)z material of sintered magnets occurs in several stages; it begins from the preparation of fine micron-sized powders, continues during the most high-temperature sintering processes and solid-solution treatment, and finishes by quenching of blanks. Owing to the efficient homogenization, the subsequent phase transformations, which occur during isothermal and stepped aging, are very difficult to be detailed by studying the material’s structure even in using modern methods and equipment owing to nano scales of structural changes. In this case, the transformations clearly manifest themselves in measuring structure-sensitive parameters of samples, in particular, the coercive force [1,2,3,4].

However, the ultimate hysteresis loops can be demonstrated by not only samples sintered from (Sm,Zr)(Co,Cu,Fe)z-based powders but also samples prepared from individual grains of coarse-crystalline ingots subjected to the analogous treatment for the high-coercivity state [5]. Within a (Sm,Zr)(Co,Cu,Fe)z-alloy grain, all phases formed during solidification and subsequent phase transformations are collinear. In this case, the absence of powder-metallurgy stages, which result in the ideally homogenized state, determines different degrees of phase transformations over the section of samples prepared directly from the cast state. This facilitates the study of details of the formation of structural components.

The following should be noted:

- Samples prepared from single grain of alloy ingot are not single crystals because of their heterophase structure. However, taking into account the collinearity of all phases comprising the samples (with the common easy magnetization axis), we call them pseudo-single-crystal samples (from here in, samples).
- We call the hysteresis loops of samples comprising an individual experimental series ultimate hysteresis loops if, during magnetic measurements, at least some samples of this series exhibit the value of (BH)_MAX_ equal to (4πJ_S_)^2^/4.

In [5], we concretized common regularities of the structure and magnetic properties of studied samples, which were prepared from individual grains of Sm_1-X_Zr_X_(Co,Cu,Fe)_Z_ alloys (with x = 0.13–0.19; z = 6.0–6.8 and different relationships of 3d-elements) in the high-coercivity state, which are related to the heterogeneity at the optical-resolution scale.

It was shown that, in optical resolutions, the structure of pseudo-single-crystal Sm_1-X_Zr_X_(Co,Cu,Fe)_Z_ samples consists of three structural components, such as A, B, and C, based on the 1:5, 2:17, and 2:7 phases, respectively. The quantitative relationships of the volume fractions of the components V_A_:V_B_:V_C_ vary over wide ranges as the chemical composition monotonically changes.

For the studied composition ranges of the Sm_1-X_Zr_X_(Co,Cu,Fe)_Z_ alloys, the increase in the relative content of 3d-elements, namely, the index z in the alloy formula is accompanied by monotonically changing the volume fractions of structural components A and B (comprising more than 90% of the total volume), i.e., the dominant volume fraction of component A changes to the dominant volume fraction of component B. The hysteretic properties of the Sm_1-X_Zr_X_(Co,Cu,Fe)_Z_ alloys subjected to complete heat-treatment cycle are strictly controlled by volume relationships of structural components A and B. In this case, the dominant volume fraction of structural component A in the alloy ensures the ultimate squareness of the hysteresis loops of samples; the equality of the fractions V_A_ = V_B_ corresponds to the combination of the high coercive force (H_CJ_) and ultimate squareness of hysteresis loop; the dominant volume fraction of component B leads to a decrease in the hysteresis loop squareness.

Finally, the increase in the relative content of zirconium (x) in the Sm_1-X_Zr_X_(Co,Cu,Fe)_Z_ alloys in the high-coercivity state is accompanied by the decrease in the precision of the dependence of coercivity on z and the shift of alloy compositions with V_A_ = V_B_ to the lower relative content of 3d-elements (i.e., z).

The aim of the present work is to get an insight into the process of the formation of high-coercivity state of pseudo-single-crystal samples of (Sm,Zr)(Co,Cu,Fe)z alloys based on the results of the studies of:

- the magnetization reversal processes of two base structural components A and B;
- the chemical composition of components A and B in samples in the high-coercivity state, the composition ranges of which ensure changing the volume fractions of these components within wide ranges;
- the peculiarities of the magnetic hardening of samples at different stages of thermal aging in accordance with the relationship of volume fractions of structural components A and B.

## 2. Materials and Methods

Table 1 gives the chemical compositions of the alloys of experimental series, which have the common formula Sm_1-X_Zr_X_(Co_1-a-b_Cu_a_Fe_b_)_Z_ and were prepared by high-frequency induction melting of individual components.

Common characteristics of the starting materials, peculiarities of the sample preparation, and experimental procedures and equipment are described in [5].

The domain structure and microstructure were studied using samples, the hysteretic characteristics of which were preliminarily completely characterized; the samples were demagnetized to zero magnetization using an external oscillating magnetic field of variable-polarity and decreasing amplitude (OFVP-demagnetized state of samples).

The major and minor hysteresis loops of the pseudo-single-crystal (Sm,Zr)(Co,Cu,Fe)z samples exhibit the ideal canonical behavior typical of materials characterized by domain-wall pinning coercivity mechanism (see Figure 1 in [5]). This allowed us to observe the domain structure by the Kerr effect method using an optical microscope and samples exposed to a magnetic field of a given value.

The 100-kOe applied magnetic field 8 ms in pulse duration was provided by capacitor-type magnetization power supply equipment (Model KCJ-3560G, Ningbo Canmag Electronics Co., Ningbo, China). The magnetic field amplitude was measured by a Mech’-2 pulse teslameter.

The quantitative analysis of the chemical composition of structural components of samples was performed using an EPMA-SEM Camebax analyzer (CAMECA, Gennevilliers, France) and SmLα, ZrLα, CoKα, FeKα, and CuKα characteristic lines and ZAF correction method.

The electron probe analysis of each sample was started from the determination of the integral chemical composition by scanning the maximally possible section area in order to control the correspondence of the sample composition to the chemical analysis data of the associated alloy. Individual structural components were studied in an analogous manner, and areas away from interface regions were analyzed. The composition was checked at individual points of structural components, and linear element scanning across boundaries of structural components was performed as well. As standards, samples of pure metals were used. The relative error of the determination of atomic concentrations of Co, Fe, Zr, Sm, and Cu did not exceed 0.5, 1.0, 4.0, 2.0, and 2.0%, respectively.

## 3. Results

### 3.1. Peculiarities of Magnetization Reversal of Main Structural Components of (Sm,Zr)(Co,Cu,Fe)z Alloys in the High-Coercivity State

**Figure 1:**
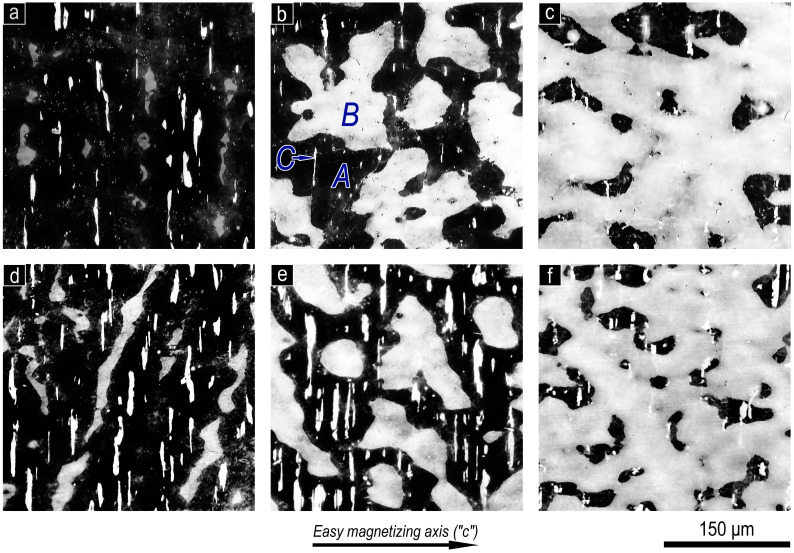
Microstructure on the prismatic plane of pseudo-single-crystal Sm_1-X_Zr_X_(Co_0.702_Cu_0.088_Fe_0.210_)z samples after complete cycle of heat-treatments for the high-coercivity state, where (**a**–**c**) x = 0.15 at z = (**a**) 6.0, (**b**) 6.5, and (**c**) 6.8 and (**d**–**f**) x = 0.19 at z = (**d**) 6.0, (**e**) 6.3, and (**f**) 6.7 (optical microscope, etching in 5% alcohol solution of HNO_3_). The easy magnetization axis is in the image plane and is strictly horizontal.

The typical microstructures of the Sm_1-X_Zr_X_(Co_0.702_Cu_0.088_Fe_0.210_)_Z_ pseudo-single-crystal samples with x = 0.15 and 0.19 were discussed in detail in [5]. Figure 1 shows the microstructures of the samples subjected to complete cycle of heat treatment for the high-coercivity state, which are given in accordance with monotonic variations of the 3d-element contents (z). The microstructures given in the central column in Figure 1 correspond to samples, the compositions of which result in the formation of approximately equal volume fractions of main structural components A and B (V_A_ and V_B_, respectively); right- and left-hand columns of micrographs correspond to samples with V_A_ >> V_B_ and V_B_ >> V_A_, respectively.

**Figure 2:**
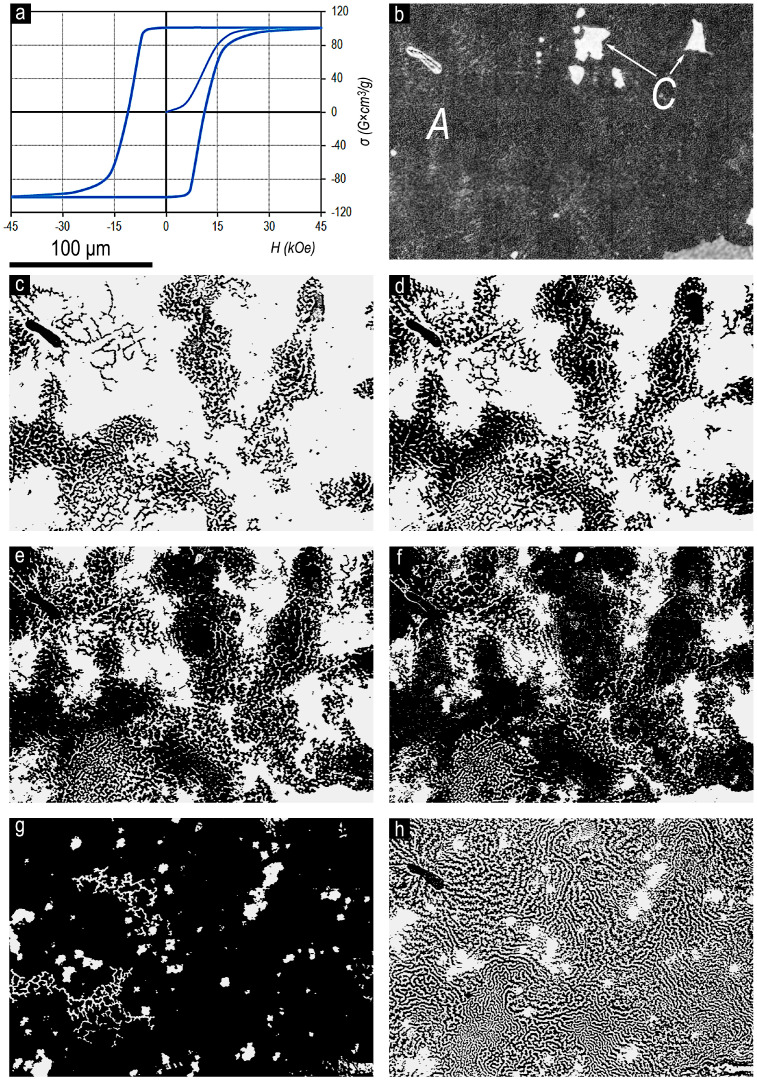
(**a**) Magnetization curve (from the OFVP-demagnetized state) and major magnetic hysteresis loop (without taking into account the demagnetizing factor N = 1/3), (**b**) microstructure on the basal plane of the pseudo-single-crystal Sm_0.85_Zr_0.15_(Co_0.702_Cu_0.088_Fe_0.210_)_6.0_ sample, (**c**–**h**) surface domain structure corresponding to area (**b**) of the sample upon progressive magnetization reversal from the magnetic saturation state with the opposite applied field (**c**) 4.5, (**d**) 6, (**e**) 8, (**f**) 10.5, and (**g**) 18 kOe; Kerr effect, easy magnetization axis is perpendicular to the image plane. (**h**) The domain structure of sample in the OFVP-demagnetized state.

Figure 2 shows the magnetization curve (from the OFVP-demagnetized state) and major hysteresis loop (without taking into account the demagnetizing factor N = 1/3) (Figure 2a); the microstructure at the basal plane of the Sm_0.85_Zr_0.15_(Co_0.702_Cu_0.088_Fe_0.210_)_6.0_ sample is also shown (Figure 2b). The surface domain structure corresponding to the section (Figure 2b) is imaged upon progressive magnetization reversal of the sample from the magnetic saturation state by external opposite-direction magnetic fields of 4.5 (Figure 2c), 6 (Figure 2d), 8 (Figure 2e), 10.5 (Figure 2f), and 18 kOe (Figure 2g). Figure 2h shows the domain structure of sample in the OFVP-demagnetized state.

**Figure 3:**
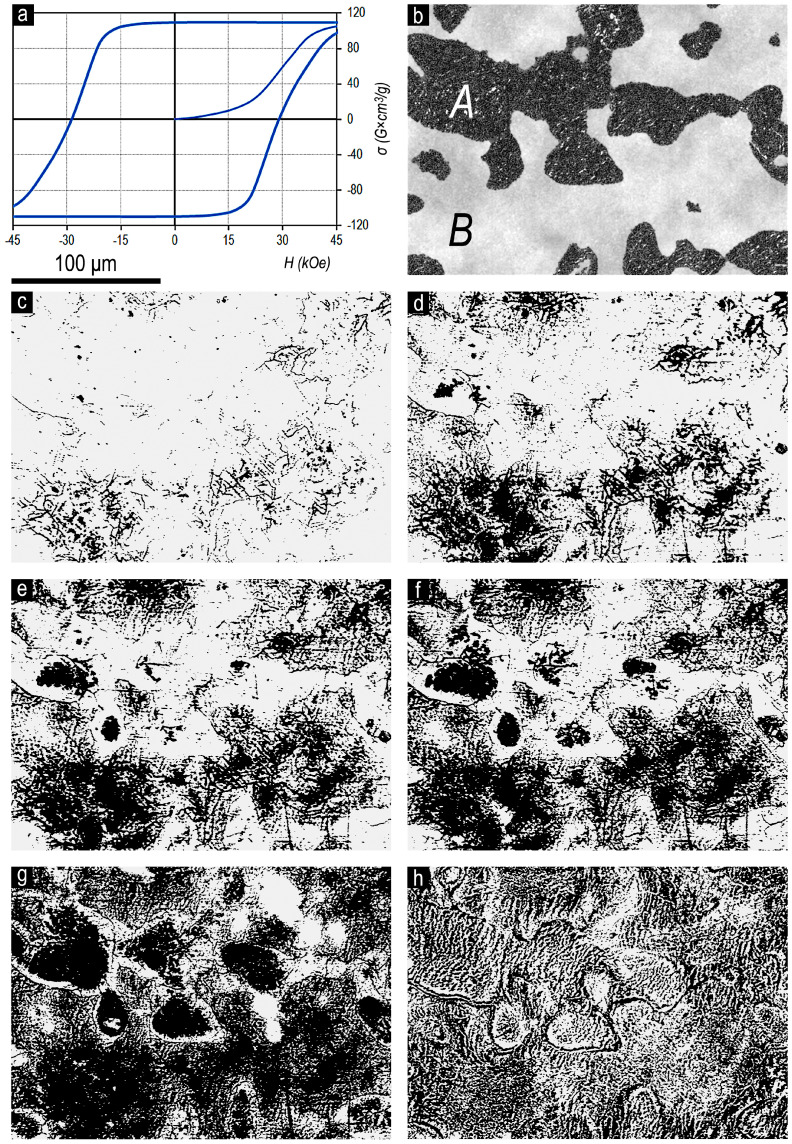
(**a**) Magnetization curve (from the OFVP-demagnetized state) and major magnetic hysteresis loop (without taking into account the demagnetizing factor N=1/3), (**b**) microstructure on the basal plane of the pseudo-single-crystal Sm_0.85_Zr_0.15_(Co_0.702_Cu_0.088_Fe_0.210_)_6.6_ sample, (**c**–**h**) surface domain structure corresponding to area (**b**) of sample upon progressive magnetization reversal from the magnetic saturation state by the opposite applied field (**c**) 10, (**d**) 15, (**e**) 17.5, (**f**) 20, and (**g**) 30 kOe; Kerr effect, easy magnetization axis is perpendicular to the image plane. (**h**) The domain structure of sample in the OFVP-demagnetized state.

In turn, Figure 3 shows the magnetization curve (from the OFVP-demagnetized state) and major hysteresis loop (without taking into account the demagnetizing factor N = 1/3) (Figure 3a) and microstructure on the basal plane of the same Sm_0.85_Zr_0.15_(Co_0.702_Cu_0.088_Fe_0.210_)_6.6_ sample (Figure 3b) and surface domain structure (Kerr effect) corresponding to the same region upon progressive magnetization reversal (from the magnetic saturation state) by an applied opposite-direction magnetic field (Figure 3c–h) of 10 (Figure 3c), 15 (Figure 3d), 17.5 (Figure 3e), 20 (Figure 3f), and 30 kOe (Figure 3g). Figure 3h shows the domain structure of sample in the OFVP-demagnetized state.

The prismatic plane of the Sm_0.85_Zr_0.15_(Co_0.702_Cu_0.088_Fe_0.210_)_6.6_ alloy sample is almost free from the structural component B. The hue of inhomogeneities in structural component A should be considered as transitional regions (A + B), i.e., a core that has not transformed into full-fledged component B. This fact more clearly manifests itself in analyzing the microstructure on the basal plane of sample having the analogous chemical composition (Figure 2b), in which the core of transition region (A+B) is presented only in the lower right core of the image. Thus, taking into account the small thickness of plates of structural component C, the transformation of the domain structure on the Sm_0.85_Zr_0.15_(Co_0.702_Cu_0.088_Fe_0.210_)_6.0_ sample surface can be considered almost completely typical of the magnetization reversal of the structural component A.

The sample given in Figure 3 is characterized by wide regions corresponding to structural components both A and B. In this case, the volume fraction of structural component B in this sample slightly exceeds that of component A (V_B_ > V_A_) [5].

Here, the consideration should be given to the fact that the common regularities of the transformation of the surface domain structure are absolutely identical for all experimental series of (Sm,Zr)(Co,Cu,Fe)z samples considered in [5] and in the present paper. In this case, within each of the structural components A and B, the character of transformation of the domain structure differs qualitatively and quantitatively for the same samples under study.

As was noted above, the studied samples are characterized by, on the one hand, the ultimate hysteresis loops and, on the other hand, substantial retaining structural inhomogeneity related to manufacturing peculiarities. Because of this, the development of the domain structure within structural components A and B occurs within wide external magnetic field ranges. Primary reverse domains nucleate in central regions of both structural components and develop toward their boundary areas (Figure 3).

The domain structure of component B in the high-coercivity state and its transformation processes in external magnetic fields are identical to the domain structure of (Sm,Zr)(Co,Cu,Fe)z magnets sintered from fine powders [3,7,8]. Upon magnetization reversal, it is characterized by the reverse domains formed from numerous centers. In this case, zigzagging domains of submicron thicknesses develop and grow only along the long axis. As the negative external magnetic field increases, the filling of section area with reverse domains occurs at the expense of adding new domains and packaging the reverse domain network rather than the increase in the domain width (Figure 3).

The high-coercivity surface domain structure of component A is qualitatively different. It is likely to be identical to that of quasi-binary Sm(Co,Cu)z and Sm(Co,Cu,Fe)z (z = 5–6) alloy samples [7,8,9]. The transformation of the domain structure of component A upon magnetization reversal occurs from a limited number of centers with the development of labyrinth domains transforming into fern-like domains that widen isotropically in all directions (Figure 2).

The maximum coercivity of domain walls is observed in the regions (A–B) being transitional from one structural component to another. This is clearly seen when comparing the locations of the boundaries of structural components A and B in Figure 3b and extended sections of samples free from reverse domains, even after exposure to a magnetization reversing field of 30 kOe in Figure 3g, as well as the stable absence of reverse domains up to relatively high magnetization reversing fields at lower right corner of image in Figure 2, which corresponds to the region with the structure (A + B). The high local coercivity of domain walls is also confirmed by the character of domain structure in the regions of the boundaries of the structural components in Figure 2 and Figure 3h in OFVP-demagnetized state of the samples.

**Figure 4:**
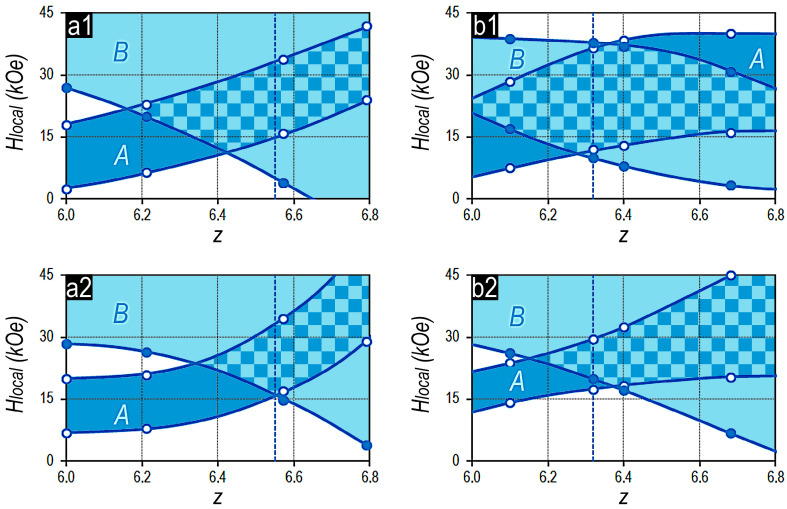
Dependence of the local coercivity of domain walls in structural components A and B upon magnetization of samples from the OFVP-demagnetized state (**a1**,**b1**) and during magnetization reversal from the saturation state (**a2**,**b2**). Data are given for pseudo-single-crystal Sm_1-X_Zr_X_(Co_0.702_Cu_0.088_Fe_0.210_)z samples with x = 0.15 (**a1,a2**) and 0.19 (**b1,b2**). Vertical dashed line indicates compositions of samples characterized by equal volume fractions of structural components A and B.

Figure 4 shows the influence of monotonic change in the ratio (3*d*)/(4*f*,4*d*), i.e., z of alloys on the ranges of local coercivity of domain walls in structural components A and B of experimental samples subjected to the full cycle of heat treatments. The data are given for the Sm_1-X_Zr_X_(Co_0.702_Cu_0.088_Fe_0.210_)_Z_ samples with x = 0.15 and 0.19 for two variants of the action of external magnetic field, namely, upon magnetization of samples from the OFVP-demagnetized state and upon magnetization reversal from the saturation state (after the action of magnetizing field of 100 kOe).

As the z value of alloy composition increases, the values of the upper and lower limits of the interval of the local coercivity of the domain walls of structural component A increase, whereas those for structural component B decrease.

It should be noted that, in the total case, taking into account all studied experimental series of alloys, the range of local coercivity of domain walls in structural component A is always narrower than that in the structural component B.

Here, it is necessary to add some comments. Initially, we understand (see Introduction) that, in the absence of powder metallurgy operations, some compositional fluctuations exist in the samples, which are due to the limited times of heat treatments and low diffusion rates typical of quinary (Sm,Zr)(Co,Cu,Fe)z system. Moreover, since we say about surface domains, under conditions of weakening the exchange between structural elements, the spread of values of local coercivity of the domain wall is substantially wider than the real intervals in the material massive. Because of this, the results mainly should be considered as common tendencies of behavior of domain wall rather than the quantitative characteristics.

In the first approximation, upon magnetization reversal, the equality of low limits of local coercivity of domain walls correspond to the equality of volume fractions of structural components A and B. As z of alloy increases more, i.e., as the volume fraction of structural component B increases, it is accompanied by the decrease in the low limit of local coercivity of domain walls in it; the worsening of the squareness of the hysteresis loop of the (Sm,Zr)(Co,Cu,Fe)z alloy samples is observed, which was noted in [5].

### 3.2. Composition Peculiarities of Structural Components of (Sm,Zr)(Co,Cu,Fe)z Alloy Samples in the High-Coercivity State

According to EPMA-SEM data for individual areas of structural components, it was found that, as the integral ratio of (3*d*)/(4*f*,4*d*) elements (z) changes, the samarium and zirconium concentrations in the main structural components remain, in average, unchanged. Therefore, the (3*d*)/(4*f*)/(4*d*) ratio in each of them (z_A_ and z_B_) is also unchanged with varying chemical composition within the experimental series of alloys.

**Figure 5:**
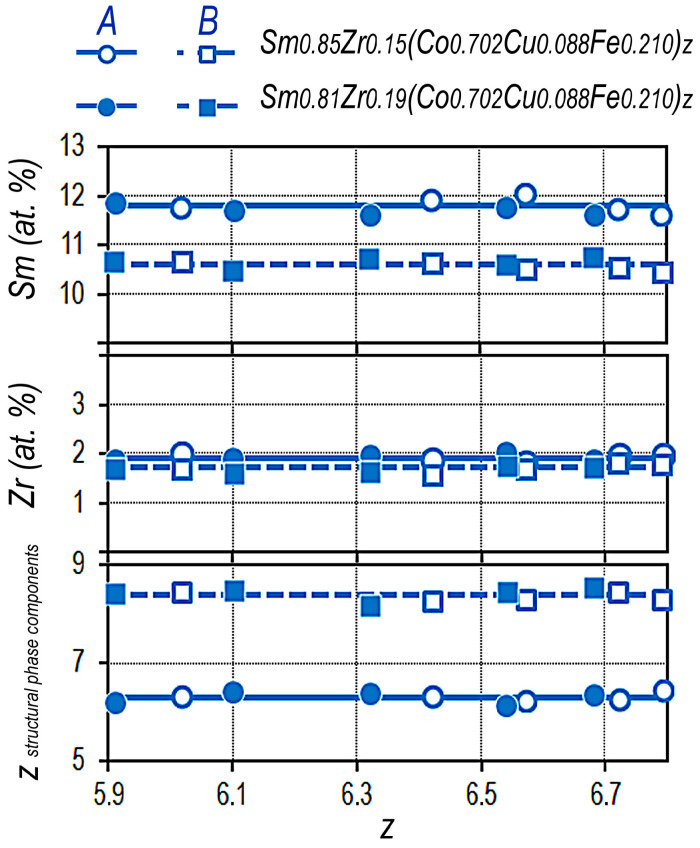
Dependencies of Sm and Zr concentrations and the ratio of (3*d*)/(4*f*,4*d*) elements of main structural components z_A_ and z_B_ on the integral z of the Sm_1-X_Zr_X_(Co_0.702_Cu_0.088_Fe_0.210_)z alloys with x = 0.15 and 0.19.

Typical dependencies of the Sm and Zr concentrations on the integral composition z of the Sm_1-X_Zr_X_(Co_0.702_Cu_0.088_Fe_0.210_)_Z_ alloys with x = 0.15 and 0.19 are shown in Figure 5. Figure 5 also shows the average ratios of (3*d*)/(4*f*)/(4*d*) elements in the main structural components z_A_ and z_B_ for the alloys of two series.

It should be noted that, since structural components A and B are formed based on the 1:5 and 2:17 phases, values z_A_ are given taking into account the formula (Sm,Zr)(Co,Cu,Fe)z_A_, whereas, the value z_B_ is given in accordance with formula Sm(Co,Cu,Fe,Zr)z_B_ and arguments available in [5], respectively.

In turn, the relationships of 3d elements in main structural components A and B in the (Sm,Zr)(Co,Cu,Fe)z samples in the high-coercivity state vary over wide ranges.

**Figure 6:**
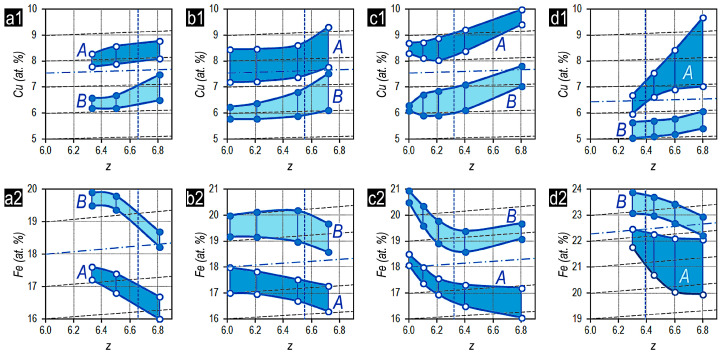
Changes of concentration ranges of Cu (**a1**–**d1**) and Fe (**a2**–**d2**) in main structural components A and B for the Sm_1-X_Zr_X_(Co_0.702_Cu_0.088_Fe_0.210_)z alloy samples with x = (**a1**,**a2**) 0.13, (**b1**,**b2**) 0.15, and (**c1**,**c2**) 0.19 and Sm_0.85_Zr_0.15_(Co_0.665_Cu_0.075_Fe_0.260_)z (**d1**,**d2**) alloy samples on the ratio z for the alloys. Dot-and-dash slanting lines show the average Cu and Fe concentrations in the alloys. Dash slanting lines show the relationship between the Cu and Fe concentrations and the z ratio of the alloys. Vertical dashed line indicates compositions of the alloys with equal volume fractions of main structural components A and B.

Figure 6 shows the concentration ranges of Cu (Figure 6a1–d1) and Fe (Figure 6a2–d2) in main structural components A and B of samples as functions of the z ratio of the experimental series: Sm_0.87_Zr_0.13_(Co_0.702_Cu_0.088_Fe_0.210_)_Z_ (a), Sm_0.85_Zr_0.15_(Co_0.702_Cu_0.088_Fe_0.210_)_Z_ (b), Sm_0.81_Zr_0.19_(Co_0.702_Cu_0.088_Fe_0.210_)_Z_ (c), andSm_0.85_Zr_0.15_(Co_0.665_Cu_0.075_Fe_0.260_)_Z_ (d). For easy analysis of results, the average Cu and Fe concentrations in the alloys are shown by dot-and-dash slanting lines. Slanting dashed lines correspond to changes in the Cu and Fe concentrations, which are due to the increase in z ratio for the alloys. The compositions of the alloys characterized by equal volume fractions of main structural components A and B are shown by vertical dashed lines.

The compositional separation of structural components A and B is clearly represented: component A substantially is enriched in Cu and depleted of Fe, whereas, component B is enriched in Fe and depleted of Cu.

In this case, the increase in the Cu concentration in structural components A and B (that is unexpected) is substantially more dynamical as compared to the calculated increase in the copper content, which is shown by additional slanting lines and is related to the increase in the fraction of 3d elements with increasing z of the alloys.

In turn, despite the increase in the Fe concentration in the integral composition of the alloy, which is related to the increase in the ratio of (3*d*)/(4*f*,4*d*) elements, i.e., z of the alloys as a whole, the substantial decrease in the Fe content is observed for all experimental series of alloys. The exception is the Fe concentration in structural component B in the alloys of series Sm_0.81_Zr_0.19_(Co_0.702_Cu_0.088_Fe_0.210_)z with z corresponding to the condition V_B_ > V_A_, at which the increase in the Fe concentration is observed, which corresponds to its increase due to the change in z.

### 3.3. Effect of the Relationship of Main Structural Components A and B on the Magnetic Hardening of the (Sm,Zr)(Co,Cu,Fe)z Alloys during Heat Treatment

**Figure 7:**
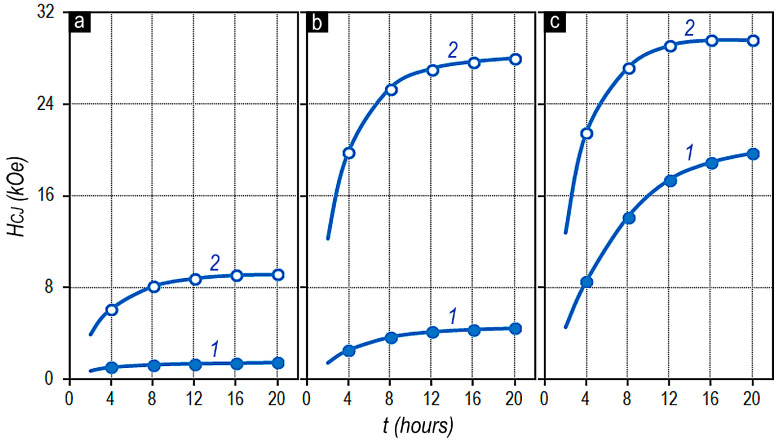
Effect of isothermal aging at 800 °C on the coercive force of experimental Sm_0.85_Zr_0.15_(Co_0.702_Cu_0.088_Fe_0.210_)_Z_ alloy samples with z = 6.0 (**a**), 6.5 (**b**) and 6.8 (**c**) subjected to subsequent (1) quenching to room temperature and (2) stepped aging (or slow cooling) to 400 °C.

Figure 7 shows the dynamics of increase in the coercive force of the pseudo-single-crystal Sm_0.85_Zr_0.15_(Co_0.702_Cu_0.088_Fe_0.210_)_Z_ samples as a function of the time of isothermal aging at 800 °C; curves 1 correspond to samples subsequently subjected to quenching and curves 2 correspond to samples subsequently subjected to stepped aging (or aging upon slow cooling) to 400 °C.

**Figure 8:**
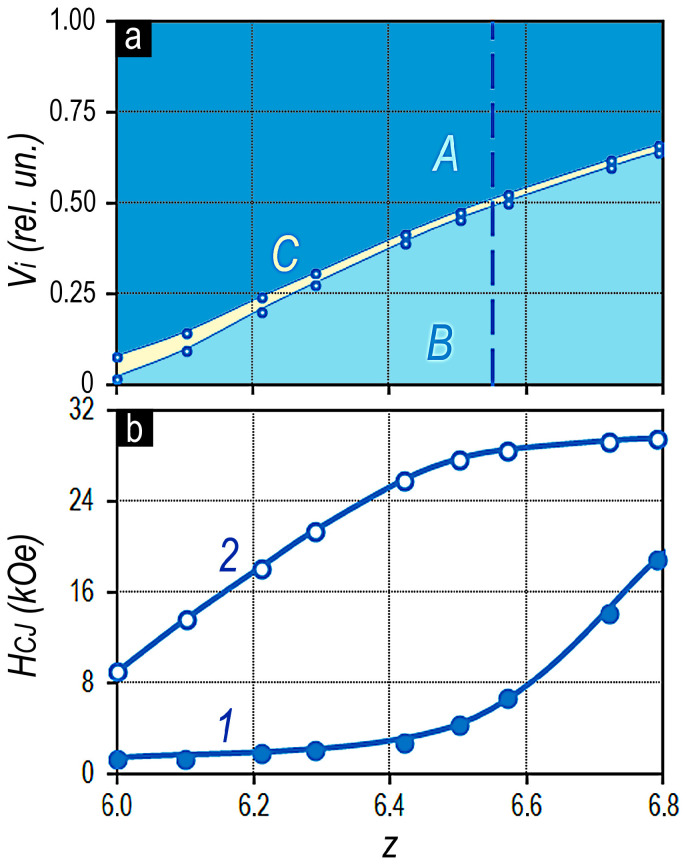
(**a**) Volume fractions of structural components A, B, and C (Vi) and (**b**) coercive force (H_CJ_) as functions of the ratio of (4*f*,4*d*)/(3*d*) elements or z of the Sm_0.85_Zr_0.15_(Co_0.702_Cu_0.088_Fe_0.210_)_Z_ alloys: 1—H_CJ_ after isothermal aging at 800 °C for 16 h and subsequent quenching and 2—H_CJ_ after stepped aging (or slow cooling) of samples to 400 °C.

Figure 8 shows relationship of volume fractions of structural components A, B, and C of the same series of alloys (Figure 8a) and corresponding dependencies of the coercive force of the alloy samples (Figure 8b), which were realized as a result of isothermal aging at 800 °C for 16 h and quenching (curve 1) and after complete cycle of heat treatment for the high-coercivity state, which include isothermal aging and stepped aging (or aging upon slow cooling) of samples to 400 °C (curve 2).

The qualitative character of the represented dependencies of the intrinsic coercive force on the aging conditions, i.e., on the completion of isothermal aging with the quenching of samples or additional stepped aging (or aging upon slow cooling) is identical for all experimental series of samples under study.

### 3.4. Effect of Relationships of Volume Fractions of Main Structural Components A and B on the Temperature Dependences of the Coercive Force (H_CJ_) of (Sm,Zr)(Co,Cu,Fe)z Alloy Samples in the High Coercivity State

**Figure 9:**
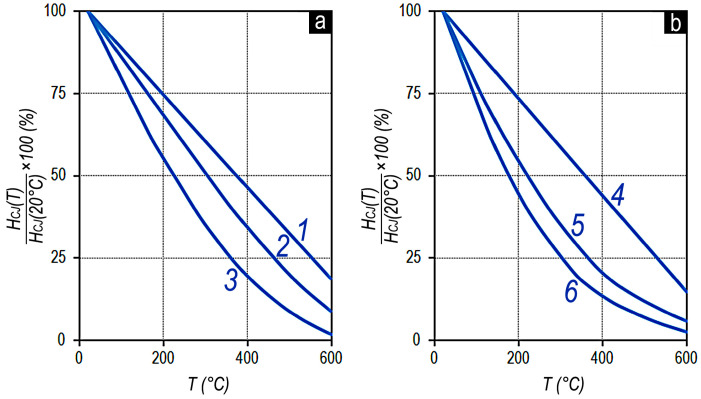
Temperature dependences of the intrinsic coercive force (H_CJ_(T)) of the Sm_1-X_Zr_X_(Co_0.702_Cu_0.088_Fe_0.210_)_Z_ alloys with x = 0.15 (**a**) and z = 6.l (1); 6.3 (2); 6.6 (3), and x = 0.19 (**b**) and z = 6.0 (4); 6.4 (5); 6.7 (6); values are reduced to H_CJ_ at 20 °C.

Figure 9 shows the effect of the sample composition on the temperature dependences of the coercive force (H_CJ_) of the high-coercivity Sm_1-X_Zr_X_(Co_0.702_Cu_0.088_Fe_0.210_)_Z_ alloy samples of two series with x = 0.15 (a) (z = 6.l (1); 6.3 (2); 6.6 (3)) and x = 0.19 (б) (z = 6.0 (4); 6.4 (5); 6.7 (6)). For convenience, the data are reduced to the H_CJ_ values at 20 °C.

Almost linear temperature dependences H_CJ_ʹ(T) correspond to the compositions with the dominant volume fraction of structural component A (V_A_ >> V_B_) (Figure 5, curves 1, 4, and partly curve 2). The sample of alloy with the dominant volume fraction of structural component B (V_A_ < V_B_) is characterized by the highest losses of intrinsic coercive force at temperatures even slightly higher room temperature (curve 6). The intermediate position (curves 3 and 5) conforms to the temperature dependences of coercivity of pseudo-single-crystal samples, the compositions of which approximately correspond to the equality of volume fractions of main structural components V_A_ ≈ V_B_ with slightly exceeding volume fraction of structural component B over that of component A (see Figure 8a and [5]).

## 4. Discussion

As was shown earlier, the microstructure of pseudo-single-crystal (Sm,Zr)(Co,Cu,Fe)z samples, which is taken with optical magnifications, is characterized by the presence of three structural components A, B, and C (Figure 1) based on the 1:5, 2:17, and 2:7 phases, respectively [5].

The structural component C is represented by plate-like precipitates arranged along the basal plane of anisotropic structural massive; its volume fraction is in the range of 0.005–0.1 (for the compositional ranges of the experimental alloy series). As the ratio of (3*d*)/(4*f*,4*d*) elements or the z index increases, the volume fraction of structural component C monotonically decreases; as the ratio of (4*d*)/(4*f*) elements or the x index increases, the volume fraction of structural component C slightly increases and depends on the ratio of 3*d* elements [5].

The sum of volume fractions of structural components A and B is 0.900–0.995 and mainly determines the hysteretic properties of samples.

The chemical compositions of structural components A and B in samples being in the high-coercivity state are characterized by substantial separation and wide ranges of contents of 3*d* elements, in particular, Fe and Cu. The compositional separation and concentration ranges given in Figure 6 result in the formation of different, in principle, fine phase structures (domain wall pining centers) in main structural components A and B. This determines (i) different morphology of domain-wall pinning centers in A and B and (ii) wide ranges of efficiency of domain-wall pinning centers within each of the components.

The domain structure of component B and processes of its transformation in applied magnetic fields are analogous to those of powder (Sm,Zr)(Co,Cu,Fe)z permanent magnets. The domain structure is characterized by the formation of thin zigzagging reverse domains of submicron width from numerous centers. As the applied magnetic field increases, no increase in the width of reverse domains occurs. The transformation of domain structure is realized at the expense of adding new generating thin domains and packing the reverse domain network at the expense of them.

The domain structure of component A is different in principle and is similar to that of quasi-binary Sm(Co,Cu)z and Sm(Co,Cu,Fe)z (z = 5–6) alloys. The number of reverse domain nucleation centers in the structural component A is lower than those in component B by orders of magnitude. As the external magnetic field increases, the transformation of domain structure occurs at the expense of the formation of labyrinth domains, which transfer into fern-like domains and grow isotropically in all directions.

The domain structure of component B indicates the similarity of its fine phase structure with the known cell-boundary-lamellar morphology of (Sm,Zr)(Co,Cu,Fe)z magnets in the high-coercivity state, which were prepared from fine powders, whereas the structural component A per se in the pseudo-single-crystal samples is the model prototype of the boundary phase between cells of the typical cell-boundary-lamellar structure of sintered (Sm,Zr)(Co,Cu,Fe)z magnets.

The volume fraction of structural component B in the alloys within the experimental series of compositions monotonically increases at the expense of almost proportional decrease in the volume fraction of component A. In the case of the highest coercivity of samples, as a result of complex isothermal and stepped aging, the increase in the volume fraction of component B is accompanied by the substantial increase in the coercive force H_CJ_ up to the moment when the volume fractions become equal V_A_ = V_B_. After that, the increase in H_CJ_ stops, and the dependence H_CJ_ = f(z) goes to “plateau” (Figure 8b, curve 2) [5].

All samples of alloys corresponding to the inequality V_A_ ≥ V_B_ are characterized by almost ultimate hysteresis loops. The magnetization reversal curves of samples are characterized by the implementation of not only the equality 4πJ_S_ = B_R_ but also the condition (BH)_MAX_ = (4πJ_S_)^2^/4 [5].

However, as the values z increase and lead to the relationship of volume fractions of structural components V_A_< V_B_, the squareness of hysteresis loop decreases, and a “step” appears in the magnetization reversal portion of the loop; the “step” increases as the difference (V_B_ − V_A_) increases [5].

As compared to the above results, the dynamics of magnetic hardening after isothermal annealing followed by quenching is almost centrally symmetrical or “mirrored” (Figure 8b).

In the first approximation, the “plateau” in the dependence H_CJ_ = f(z) corresponds to the z values determining the condition V_A_ > V_B_ and is characterized by the lowest values of the coercive force. The intense increase in H_CJ_ is observed in the composition range of alloys characterized by dominant fraction of structural component B, i.e., V_A_ < V_B_.

The character of the dependences of the coercive force of experimental samples (curves 1 in Figure 7a and Figure 8 shows that, in samples characterized by the dominant volume fraction of component A, efficient domain-wall pinning centers are absent after quenching from the isothermal aging temperature, i.e., no phase transformations resulting in the formation of the pinning centers in the structural component A occur at the isothermal aging temperatures.

On the contrary, as a result of stepped aging, sufficiently efficient pinning centers form within structural component A, which lead to the ultimate hysteresis loops of experimental samples. This is likely to be related to phase transformations occurred within structural component A in a temperature range of 800–400 °C.

In fact, the substantial increase in the coercive force of samples with V_A_ < V_B_ quenched from the isothermal aging temperature indicates the phase transformations in structural component A.

Diffusion processes occurred upon isothermal aging of (Sm,Zr)(Co,Cu,Fe)z alloys ensure not only the development of cellular phase morphology but also the formation of chemical separation between the structural components. The redistribution processes of elements between structural components upon isothermal treatment are not so efficient as compared to those occurred upon stepped aging; nevertheless, they result in the substantial compositional separation [10,11,12]. In particular, the cellular 2:17 phase in structural component B and, in whole, structural component B are donors of Cu and are enriched in Fe, whereas the boundary phase B and structural component A, vice versa, give back Fe and uptake Cu (Figure 6).

At the end of isothermal aging, in particular, under prolonged holding conditions, main structural components A and B in the (Sm,Zr)(Co,Cu,Fe)z samples with z from 6.0 to 6.8 are supersaturated to a different degree with corresponding components because of:

- the same level of concentrations of main elements in the integral composition of the alloy after solid solution heat treatment;
- directional diffusion of elements between structural components formed based on different phases;
- wide ranges of variations of the volume fractions of components A and B in accordance with z of alloy.

As is known, just the presence of copper in the alloy composition ensures the generation of efficient pinning centers in the Sm(Co,Cu)z and Sm(Co,Cu,Fe)z alloys and in the boundary phase of (Sm,Zr)(Co,Cu,Fe)z compositions [2,3,9,11,12].

It is obvious that the supersaturation of component A in experimental samples with V_B_ > V_A_ reaches the level, at which the phase transformation ensuring the formation of efficient pinning centers occurs even under conditions of quenching of samples after isothermal treatment. In this case, the higher the volume fraction of component B in the alloy, i.e., the higher the difference (V_B_ − V_A_), the higher the supersaturation of component A with copper (Figure 6) and the more efficient the domain-wall pinning centers after cooling (Figure 8b, curve 1). This is also confirmed by the intensity of increase in ranges of the local coercivity of domain walls in the structural component A with increasing ratio z of the alloy, i.e., with increasing volume fraction of component B being the donor of Cu for component A decreasing in volume.

The above considerations show that the statement about the fact that the final composition of phases of the fine structure of the (Sm,Zr)(Co,Cu,Fe)z alloys upon heat treatment for the high-coercivity state forms only in the course of isothermal aging and the fact that phase transformations are absent at the stepped stage of treatment (or aging upon slow cooling) is untenable.

Upon isothermal treatment of sintered (Sm,Zr)(Co,Cu,Fe)z magnets, the solid solution based on the disordered 1:7H phase decomposes with the formation of cellular structure. In our model pseudo-single-crystal samples of alloys, the structure with the cellular morphology forms within component B. Structural component A is the complete analog and sequential continuation of boundary network coming from the component B. Therefore, the structure of component A is analogous to the structure of boundaries between cells and is formed by sequential alternating layers of phases 2:7R and 5:19H along the c axis [11,13,14]. In turn, due to intense diffusion during stepwise aging (or slow cooling), the final high-coercivity structure of the alloy is formed based on 2:17R + 1:5H phases.

In (Sm,Zr)(Co,Cu,Fe)z alloy samples with the ratio of (4f,4d)/(3d) elements (z) above a certain limit, the dominant volume fraction of structural component B leads to the high degree of supersaturation of structural component A with copper. This predetermines the occurrence of transformation 2:7R + 5:19H + [Cu] → 1:5H upon quenching without stepped aging (or slow cooling) in a temperature range of 800–400 °C.

Because of the short time of quenching, the phase transformation in component A and boundary phase B is likely to depend on the ratio of their volume fractions and is characterized by different degree of completion that is inverse V_B_/V_A_. Because of this, the magnetization reversal curves of samples with V_B_ > V_A_ quenched after isothermal aging are characterized by lower hysteretic parameters as compared to those of samples with V_A_ ≥ V_B_ after stepped aging (or slow cooling), which ensures the completion of phase transformations.

The reversibility of phase transformations occurred in a temperature range of 800–400 °C easily explains the know phenomenon “return” of coercivity upon short-time heating of sintered magnets and model samples based on alloys with low ratios (4f,4d)/(3d) (or low z in (Sm,Zr)(Co,Cu,Fe)z) to the isothermal aging temperature.

Taking into account the reported peculiarities of experimental (Sm,Zr)(Co,Cu,Fe)z alloys in the high-coercivity state, the temperature dependences of the coercivity (Figure 9) can be easily described in terms of the “repulsive-attractive pinning” model described in detail by Goll et al. [11].

It was shown that, with increasing Sm (in our case, (Sm,Zr)) content or decreasing z values (6.8→6.0), the quinary system (Sm,Zr)(Co,Cu,Fe)z shows a decrease in the temperature coefficient (dHc/dT)/Hc. This behavior is rigorously related to the phase structure and the temperature dependence of the phases’ material parameters as J_S_, K_1_, and A. The decrease in z values in (Sm,Zr)(Co,Cu,Fe)z alloys, in accordance with our results, is unambiguously associated with the increase of the volume fraction of structural component A in them. Therefore, samples with the compositions corresponding to the condition V_A_ > V_B_ exhibit lower temperature coefficients of the coercive force, which appears in the flatter dependence H_C_(T).

## 5. Conclusions

The present study was performed to widen the objective picture about the formation of the structure and magnetic properties of (Sm,Zr)(Co,Cu,Fe)z alloys with the compositions that show promise for manufacturing high-coercivity permanent magnets; the present paper is the logical continuation of work [5].

As a result of studies of magnetization reversal processes, the chemical composition of two main structural components A and B of pseudo-single-crystal (Sm,Zr)(Co,Cu,Fe)z alloy samples, and the peculiarities of heat-treatment-induced magnetic hardening of the samples in accordance with the relationship of volume fractions of components A and B, the following conclusions can be inferred.

1. The heat treatment for the high-coercivity state leads to the substantial separation of two main structural components (A and B) in the chemical composition; the separation is caused by different temperature dependencies of the solubility of 3*d* elements in the phases being the basis for these structural components (1:5 and 2:17, respectively).
2. The separation of the structural components in the chemical composition predetermines the formation of different domain-wall pinning centers (thin phase structure), which determines qualitative and quantitative differences in the development of surface domain structures in structural components A and B upon magnetization reversal.
3. The surface domain structure of component B is the network of reverse zigzagging submicron-thick domains, which arise from numerous centers. As the magnetization reversing magnetic field increases, the magnetization reversal of B occurs at the expense of appearance of new domains that progressively fill the reverse-domain network rather than increasing the thickness of the domains.
4. The surface domain structure of component A is analogous to that of Sm(Co,Cu,)x and Sm(Co,Cu,Fe)x alloy samples (x = 5–6). Its development upon magnetization reversal occurs from a limited number of centers at the expense of the formation of reverse labyrinth domains, which transfer into fern-like domains growing isotropically in all directions.
5. The domain structure of component B in the pseudo-single-crystal samples indicates that the morphology of its fine phase structure is identical to the structure of sintered (Sm,Zr)(Co,Cu,Fe)z magnets, which comprises cellular, boundary, and plate-like phases. In turn, the phase structure of component A is the model prototype of the boundary phase in sintered magnets.
6. There is direct interrelation between the chemical composition of samples, relationship of volume fractions of main components (A and B) in the structure of samples, and development of the coercive force as a result of magnetic hardening upon isothermal aging followed by quenching or upon isothermal and stepped heat treatments.
7. The cellular morphology in the alloy structure is formed in the course of isothermal aging, whereas the final phase compositions of component A and boundary phase B are formed in a temperature range from isothermal aging temperature to 400 °C upon stepped (slow) cooling or quenching.
8. The propensity of chemical compositions of component A and component’s B boundary to phase transformations after the completion of isothermal aging (quenching or stepped aging) and degree of completion of these transformations directly depend on the relationship of volume fractions of component A and B and the (4*f*-,4*d*-)/(3*d*-) element ratio (i.e., z) in the (Sm,Zr)(Co,Cu,Fe)z alloy. In turn, the degree of completion of phase transformations determines the final hysteretic properties of samples.
9. The viewpoint that the formation of the final phase composition in the (Sm,Zr)(Co,Cu,Fe)z alloy occurs only in the course of isothermal stage of heat treatment and that phase transformations are absent at the stage of stepped aging (or slow cooling) is inaccurate.

## Figures and Tables

**Table 1:** Chemical compositions of experimental Sm_1-X_Zr_X_(Co_1-a-b_Cu_a_Fe_b_)z alloys (x, a, and b are atomic fractions).

Series No.	x	a	b	z
1	0.13	0.088	0.210	6.0–6.8
2	0.15	0.088	0.210	6.0–6.8
3	0.17	0.088	0.210	6.0–6.8
4	0.19	0.088	0.210	6.0–6.8
5	0.15	0.075	0.260	6.0–6.8
